# Optimization of Extraction Conditions and Cytotoxic Activity of Rapanone in Comparison to Its Homologue, Embelin

**DOI:** 10.3390/molecules27227912

**Published:** 2022-11-16

**Authors:** Dagmara Wróbel-Biedrawa, Agnieszka Galanty, Paweł Zagrodzki, Irma Podolak

**Affiliations:** 1 Department of Pharmacognosy, Pharmaceutical Faculty, Medical College, Jagiellonian University, Medyczna 9, 30-688 Cracow, Poland; 2 Department of Food Chemistry and Nutrition, Pharmaceutical Faculty, Medical College, Jagiellonian University, Medyczna 9, 30-688 Cracow, Poland

**Keywords:** rapanone, embelin, cytotoxic, extraction, optimization, cancer

## Abstract

Rapanone is a plant-derived simple alkyl-dihydroxybenzoquinone structurally close to embelin, a well-known cytotoxic agent. The pharmacological characterization of rapanone is still incomplete, and to fill the data gap, a good source for its acquisition is required to conduct further research. This study aimed to optimize the conditions for the extraction of rapanone from the leaves of white-berried *Ardisia crenata* Sims. For this purpose, three methods were employed: heat reflux (HRE), shaking (SE), and ultrasound-assisted extraction (UAE), and such parameters as the extraction time, solvent, and the number of extractions from the same sample were set as experimental variables. Furthermore, cytotoxic activity toward prostate cancer, thyroid cancer, and colorectal carcinoma cell lines was investigated and compared with doxorubicin and embelin. The most effective and economical method for the extraction of rapanone was shown to be 20 min UAE with ethyl acetate or chloroform. Rapanone exhibited high cytotoxic activity against PC3 (IC_50_ = 6.50 μg/mL), Du145 (IC_50_ = 7.68 μg/mL), FTC133 (IC_50_ = 6.01 μg/mL), 8505C (IC_50_ = 7.84 μg/mL), and Caco-2 (IC_50_ = 8.79 μg/mL) cell lines after 24 h and against the HT29 cell line after 48 h (IC_50_ = 11.67 μg/mL). Furthermore, it revealed a more favorable safety profile than either its homologue, embelin, or doxorubicin. The set of optimal extraction parameters obtained may be utilized for scientific and industrial purposes to achieve the best rapanone yield. Moreover, this benzoquinone revealed a high cytotoxic activity with good selectivity.

## 1. Introduction

**Figure 1:**
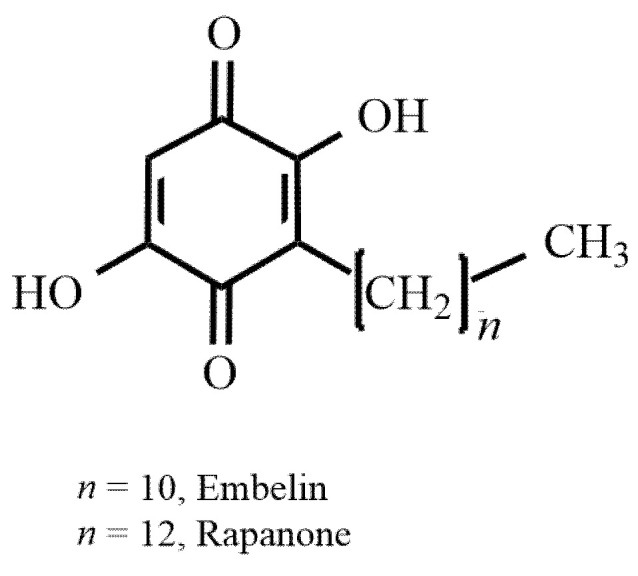
Structure of rapanone and embelin.

Rapanone (Figure 1) represents simple alkyl-dihydroxybenzoquinones that occur naturally in plants. Hydroxybenzoquinone derivatives are considered typical secondary metabolites for some species belonging to the *Primulaceae* family, especially those that were previously classified to the *Myrsinaceae*. Rapanone, along with its structural homologue, embelin, is often concomitant in some plant species, e.g., *Ardisia crenata* Sims [1], *Lysimachia punctata* [2], *Myrsine guianensis* [3]. These two alkyl-dihydroxybenzoquinones differ structurally in only two carbon atoms in the side chain (Figure 1). Most published reports refer to embelin, which is an active ingredient of *Embelia ribes*, a well-known Ayurvedic medicinal plant used as a wound healer, anti-inflammatory, antimicrobial, or contraceptive agent [4,5]. Recent studies not only supported some of the traditional uses of the plant in relation to embelin, but also showed new directions of its pharmacological activity with potential applications: cytotoxic, anti-diabetic, anxiolytic, antidepressive, neuroprotective, and cardioprotective [6,7,8]. The compound was also shown to be an XIAP (X-linked inhibitor of apoptosis protein) inhibitor [9]. The pharmacological properties of rapanone, in turn, are poorly investigated in comparison to those of embelin, although the similarity of their structures allows us to assume a high biological potential of rapanone, as well. The results of recent studies revealed anti-inflammatory [10,11], antifertility [3], cytotoxic [11,12,13,14], and antioxidant [11,15] activity.

Rapanone was isolated for the first time from *Rapanea maximowiczii* (Koidz) by Kawamura and Hokoku in 1937, just five years after embelin was evaluated as an active compound of *E. ribes* [16]. As mentioned above, both compounds often occur together, with embelin as the predominant benzoquinone constituent. Our team found that *A. crenata* Sims may be a good source to obtain rapanone solely [1]. As with other phytochemicals, the yield greatly depends on the applied extraction procedure. Although there are a couple of studies optimizing the extraction conditions for embelin from *E. ribes* [17,18], no data on the relationship between the selection of extraction parameters and the efficiency of the process are available for rapanone. Taking this into account, we aimed to optimize the extraction conditions of this benzoquinone from the leaves of the white-berried variety of *A. crenata* Sims.

In 2020, almost 20% of all deaths worldwide were caused by cancer [19]. Among the different types of cancer, colon and rectum cancers, as well as prostate cancers, are the most common [19]. Furthermore, the incidence of thyroid cancer is increasing and recently has become the fifth most common neoplastic change in the USA [20]. Given these alarming statistics, it is clear that preventive measures are not enough and the need for more effective and safer pharmacotherapeutics is still unmet. That motivates a continued search for new therapeutic options. According to the investigations performed previously and the structural similarities of rapanone and embelin, we assumed that the former should also possess interesting cytotoxic activity. 

**Figure 2:**
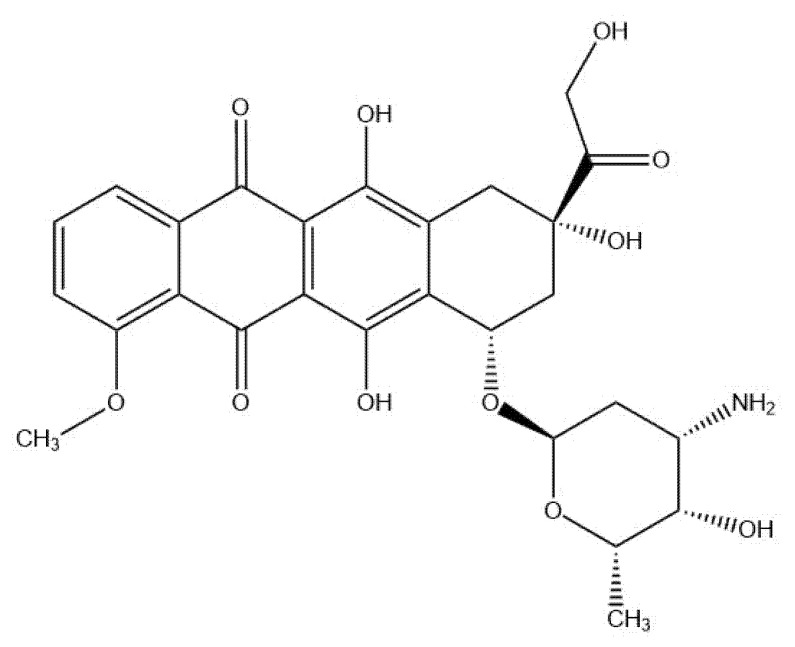
Structure of doxorubicin.

Taking the abovementioned into account, the aims of our study were to optimize the extraction conditions of rapanone and to discuss the influence of different variables of the extraction process on the yield of rapanone as well as to evaluate its cytotoxic potential by checking the impact on cancerous cell lines from the prostate, thyroid, and gastrointestinal panel. The activity of rapanone was compared to the reference drug, doxorubicin, which is a quinone derivative (Figure 2), as well as to embelin, as a structural homologue. Doxorubicin is a cytostatic drug used in chemotherapy of many types of cancers, for example, in prostate, thyroid, gastric, or hepatic cancer. Furthermore, it is widely used as a standard in testing substances for cytotoxic potential.

## 2. Results

### 2.1. Optimization of Rapanone Extraction from Plant Material 

To optimize the extraction conditions, we have checked the influence of the following variables in our study: the method (heat reflux extraction (HRE), shaking (SE), and ultrasound-assisted extraction (UAE)), the extraction time (30, 60, and 120 min (HRE, SE), or 10, 20, and 30 min (UAE)), the number of extractions from the same plant material sample (1–3 repetitions of extraction from the same sample and every repetition was conducted with the new portion of the solvent), and the solvent (chloroform, ethyl acetate, and acetone) (Table 1). As a result, we obtained the yields of rapanone (counted as milligrams of rapanone per 1 g of dried plant material) in every combination of parameters (Table 2).

Based on the analysis carried out separately in each experimental system (HRE, SE, and UAE), it could be concluded that the only factors significantly affecting the amount of rapanone in the extracts obtained in the UAE system were the linear (*p* = 0.000) and quadratic effects (*p* = 0.000) related to the type of solvent. Their corresponding regression coefficients in the multinomial model, provided in the ANOVA procedure, for the normalized values of the input parameters, were −8.84 and −5.66, respectively. The constant value in the same model was 16.32 and R^2^ = 0.909. In the other two systems, the factors significantly influencing the amount of rapanone were: the square effect related to the solvent and the linear effect related to the extraction time (system HRE), and both the square and linear effects related to the solvent and the linear effect related to the time (system SE) (detailed data were not shown as the results obtained in these systems were significantly lower than in the UAE system).

The results obtained for the various experimental systems are presented in Table 2.

The rapanone content in the extracts obtained by different methods varied from a close to zero amount up to 21.39 ± 1.21 mg per 1 g of dry weight (d.w.) of the plant material (white-berried A. crenata leaves) (median = 21.06 mg/g). The highest content was determined after ultrasound-assisted extraction, performed once for 20 min, using ethyl acetate as a solvent (Table 1). Of these highest results, four revealed the largest number of significant differences from the rest of the results: 13U, 4U, 7U, and 14U. They were all obtained by the UAE, with chloroform or ethyl acetate; however, two extractions demanded a longer extraction time (7U) or repeated extraction (14U), so they required more time and solvent investment. Taking that into account, extractions 4U and 13U seem to be more favorable.

Generally, the lowest rapanone content was obtained for the acetone extracts in the three methods used to prepare the extracts (Table 2); however, among them, the HRE-prepared extracts gave the best results, as only in this case the rapanone content was noticeable.

### 2.2. Cytotoxic Activity of Rapanone and Embelin

Our study revealed a high cytotoxic activity of rapanone with low, that is, below 10 μg/mL, IC_50_ values for almost all cancer cell lines tested (except HT29, which was still below 20 μg/mL) (Table 3, Table 4 and Table 5). For embelin, all IC_50_ values were below 25 μg/mL. In the case of thyroid cancer cell lines, the values were higher than 10 μg/mL, which showed a weaker cytotoxic effect compared to rapanone (Table 5). Interestingly, doxorubicin activity was low in metastatic grade IV prostate cancer PC3 and thyroid cancer 8505C, with IC_50_ values greater than 50 and 40 μg/mL, respectively (Table 3 and Table 5). It should be noted that in the case of rapanone, after 24 h the IC_50_ values were, respectively, 6.50 and 7.84 μg/mL, and for embelin 9.27 and 18.86 μg/mL. 

Both rapanone and embelin revealed high cytotoxicity in Caco-2 cells, as well as the other cell line from the gastrointestinal panel, HT29; however, the activity of both compounds was still lower compared to doxorubicin (Table 4).

**Figure 3:**
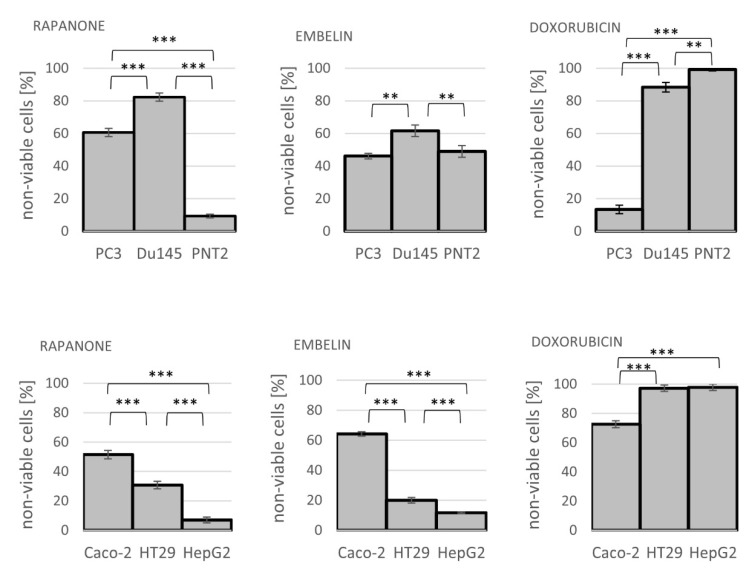
Cytotoxic activity of rapanone, embelin, and doxorubicin at 10 μg/mL towards cell lines of prostate and gastrointestinal panels (see Material and Methods section for cell line descriptions); ** *p* < 0.01, *** *p* < 0.001.

Notable is that the activity of rapanone in non-neoplastic cell lines, prostate epithelial cells, PNT2, and hepatocellular carcinoma, HepG2 was always lower than for cancerous cells, with IC_50_ values greater than 10 μg/mL, at least twice as high than for cancer cell lines (Table 3 and Table 4). After 24 h of incubation at 10 μg/mL of rapanone, the mean percentage of dead Du145 cells was nine times higher than for PNT2 (82.33% vs. 9.30%), while for embelin it was 61.67% vs. 49.00% and the reference, doxorubicin, was even more toxic to normal cells, killing almost all of them (88.38% vs. 99.21%) (Figure 3). 

Noticeably, increasing the incubation time (24 and 48 h) resulted in a decrease in IC_50_ values for rapanone and embelin (Table 3, Table 4 and Table 5), indicating a higher number of dead cells with longer exposure to each benzoquinone.

## 3. Discussion

Rapanone is a plant-derived benzoquinone that possesses promising biological potential. To investigate its pharmacological activities more thoroughly, it is vital to provide the substance in a proper amount. Our previous studies indicate that the leaves of white-berried *A. crenata* Sims, a plant that can be cultivated under greenhouse conditions, may serve as a good source for the isolation of rapanone [1,11]. In search of an effective extraction procedure, we decided to explore this issue in more detail. 

To define the most efficient conditions for the extraction of rapanone, we chose variable parameters for the study based on the literature and our own experience. As data on rapanone isolation are limited, the literature search focused on embelin, a structural homologue of the former (Figure 1). The first isolation of embelin was made in 1932 by Paranjpe and Gokhale from *E. ribes* fruits [16]. Subsequently, it was isolated from *n*-hexane extracts of *E. ribes* berries (obtained by cold maceration or heat extraction) [21,22,23,24], dichloromethane extract [25], ethanol extract [26], methanol extract of leaves of *E. ribes* [27,28], or from petroleum ether and chloroform extracts of *M. africana* berries (as reported by Kiprono [16] based on previous works).

In the available literature, in which the isolation of rapanone was reported, this compound was derived from ethyl acetate extracts obtained by simple maceration, e.g., from the stem bark of *Connarus venezuelanus* Baill [14,15], or the root bark of *C. suberosus* [29]. Furthermore, chloroform [2] or petroleum ether [3,30] was used for further fractionation of these extracts.

Taking these data into account, we selected five solvents: *n*-hexane, chloroform (as it is often recognized to possess separation properties better than dichloromethane), ethyl acetate, acetone, and methanol. A preliminary study (data not published) revealed that chloroform, ethyl acetate, and acetone had the best extraction ratio, so these were chosen for further experiments. Other optimized parameters were the method (heat reflux extraction (HRE), shaking (SE), and ultrasound-assisted extraction (UAE)), the extraction time (30, 60, and 120 min (HRE, SE), or 10, 20, and 30 min (UAE)), and the number of extractions from the same plant material sample (1–3 repetitions of extraction from the same sample and every repetition was conducted with the new portion of the solvent). The volume of the solvent (20 mL) and the degree of fragmentation (fine grounded) were experimentally fixed by us (data not shown).

Generally, our study has shown that chloroform and ethyl acetate appear to have better rapanone-extracting properties than acetone. However, in the case of HRE and SE, which are conventional methods but still commonly used in laboratories due to their simplicity, in most variants of extraction, the level of rapanone in acetone extracts was similar to that of chloroform ones. Keeping this in mind, even though chloroform is used to extract benzoquinones from different plant materials [1,16], in the case of *A. crenata* leaves, this solvent may not be the best choice for conventional techniques. In our experiment, only in the case of the UAE, chloroform was a significantly more efficient extractant compared to other solvents tested. Shaking, in turn, which requires very mild conditions of the process (see the Materials and Methods section), is the safest method for tissues and active substances because the temperature is low, and the extraction is based on simple diffusion. In this case, only using ethyl acetate as an extractant, the mean rapanone content was significantly high, but, contrary to HRE, during the shortest extraction (30 min repeated twice or three times). 

The conventional liquid-solid extraction methods (HRE, SE) are time-consuming, laborious, and require a lot of solvents to obtain the expected yield of the substance [31], whereas sonication (UAE) represents a green approach for the process, consuming less time, solvent, and work. Its higher effectiveness is due to very locally generated high pressure and temperature, causing a faster and better penetration into plant tissue, as well as the transportation of compounds [32]. 

In our study, when the UAE was applied, the highest rapanone content was achieved for chloroform and ethyl acetate. Interestingly, increasing the number of extraction repetitions did not bring any significant benefits. Furthermore, in a couple of cases, a decreasing trend in terms of rapanone content was observed in the variant of three repetitions for two longer extraction times (20 and 30 min). Generally, in the UAE, the extraction efficiency is highest at the beginning of the sonication process [32]. Exceeding that optimal time can result in the degradation of the cellular structures of the plant material or simply limiting the yield of the process, which is no longer beneficial. For *A. crenata* leaves, 20 or30 min UAE, once or twice, is enough to obtain optimal efficiency with either chloroform or ethyl acetate. As there was no significant gain in increasing the number of extractions or extending the time over 20 min, it is more economical to choose more time-, solvent- and energy-sparing parameters. Good results were obtained in a 10 min process with chloroform. Here, a trend of increasing the content of rapanone with an increase in the number of repetitions was observed. However, repeating the extraction from the same sample requires additional portions of the solvents, which makes this option less favorable. 

All in all, it seems that chloroform and ethyl acetate are both good extractants for rapanone from the leaves of *A. crenata*. The best results were obtained by the UAE method, in which the shortest time (20–30 min) was required and the volume of solvent used was low. An alternative may be maceration with shaking for 30 min with ethyl acetate, but twice or three times, which is definitely more time- and solvent-consuming. The classical heat reflux method (HRE) seems not to be an economically attractive option, as it involved much more time and repetitions of the process to obtain the best yield. Extraction with ethyl acetate in all three methods resulted in a significantly high rapanone content. 

The second part of our experiment concerned the assessment of the cytotoxic activity of rapanone. Its potential was compared with embelin, a structural homologue of rapanone, which is a known cytotoxic agent, and an XIAP inhibitor [9]. Embelin activity has been previously reported not only in vitro but also in vivo models (see recent reviews [7,33,34]). Doxorubicin was used as a reference because it is a cytostatic, commonly used in the investigation of potential anticancer agents, and a clinical chemotherapeutic for the treatment of different cancers, e.g., prostate, thyroid, gastric, or hepatic. Interestingly, its structure contains a quinone moiety; however, instead of the simple benzene configuration with two carbonyl groups, as in the case of rapanone or embelin, there is a more complex configuration of tetracene (Figure 1 and Figure 2).

Previously, we reported that rapanone was a selective cytotoxic agent against the melanoma cell line, WM793 [11]. Other studies showed its cytotoxic potential towards several cancer cell lines: Hep-2 (larynx cancer), MCF-7 breast cancer), MKN-45 (gastric cancer), SPC-212 (human mesothelioma), A549 (lung carcinoma), and weak activity for DLD-1 (colorectal adenocarcinoma) [12,13]. In the current study, we evaluated the in vitro cytotoxic potential of rapanone against cancers that are an overwhelming therapeutic challenge affecting a high percentage of the population. The compound was found to be cytotoxic against all cancer cell lines studied: prostate, Du145 and PC3, thyroid, FTC133 and 8505C, colorectal, Caco-2, and HT29, in a dose- and time-dependent manner. Our study not only proved the in vitro anti-cancer potential of rapanone itself, but also demonstrated some interesting differences between its activity and those of embelin and doxorubicin. Doxorubicin is known to be a very potent cytotoxic agent; however, in our study, it was not in the case of 8505C thyroid cancer or PC3 prostate cancer cell lines. Other authors reported IC_50_ of doxorubicin towards PC3 lines at 0.51 µM [35] which still was about 20 times lower activity than towards Du145 [36]. Nevertheless, the doxorubicin resistance of PC3 or 8505C cell lines is a well-known phenomenon [37,38]. Rapanone, in contrast, revealed a high anti-cancer potency in both cases, which was even slightly stronger than that of embelin. Previously, embelin was shown to inhibit the proliferation of castration-resistant prostate cancer cells from PC3 and Du145 lines with an IC_50_ value of 13.6 μM and 21.3 μM, respectively [39]. It induced G1 cycle arrest and apoptosis. The Akt/NF-κB/survivin signaling pathway was found to be involved in the mechanism of action of this benzoquinone [39]. Park et al. [40] also indicated that embelin caused apoptosis in the PC3 cell line. Furthermore, it was shown to participate in an inhibition of Akt and GSK-3β activation in prostate cancer cells. Kim et al. [41], in turn, demonstrated an involvement of the Akt/mTOR/S6K1 signaling cascade in the mechanism of pro-apoptotic action of embelin. Heo et al. [42] indicated the STAT3 pathway as a target of a possible mechanism assigned to benzoquinone anticancer activity towards the Du145 cell line.

Furthermore, rapanone activity was similar against both metastatic prostate cancers: Du145 and PC3. It should be noted that PC3 is a grade IV prostate carcinoma, very differentiated from normal cells, and highly aggressive. This is the first report on the antiprostate cancer potential of rapanone, and such a high activity of rapanone against this malignant type of cancer is a tremendously hopeful and promising result. 

In addition, rapanone was highly active in both thyroid cancer cell lines, FTC133 and 8505C. This is the first report on its antithyroid-cancer potential. Again, its activity seemed to be even better than that of embelin. The latter was previously shown to induce apoptosis in different thyroid cancer cell lines: BRAF V600E-mutant KTC-1 and 8505C, and wild-type FTC-133 and CAL-62 [43]. Furthermore, it inhibited the cell growth of the PTC (papillary thyroid carcinoma) cell line, causing apoptosis, and induced tumor regression in the PTC xenograft in nude mice [44].

In the case of gastrointestinal adenocarcinoma, the Caco-2 cell line, rapanone was slightly less active than embelin (IC_50_ values: 8.76 vs. 6.12 μg/mL), but had a better effect on the HT29 cell line (IC_50_ values: 16.91 vs. 24.70 μg/mL). However, the IC_50_ value for rapanone obtained in our study is slightly higher than that reported by Cordero et al. [12], 29.4 μM, which is equal to 9.3 μg/mL. Embelin, in turn, induced oxidative stress (lipid peroxidation, reduction in reduced glutathione and glutathione S-transferase) in HT29 cells, causing their apoptosis in the study conducted by Sumalatha [45]. The IC_50_ calculated by that team was slightly higher than in our experiment, that is 35 µg/mL. 

Excitingly, in our study, rapanone showed a good selectivity profile that was more beneficial than that of both doxorubicin and embelin. Embelin was only a bit less toxic to normal PNT2 cells than to cancerous ones, PC3 and Du145, while doxorubicin already at 10 μg/mL killed almost all normal cells, whereas cancer cells lines exhibited higher resistance towards the reference cytostatic. However, in the case of HepG2 cells, chosen as the second non-neoplastic cell line in our study, Kuete et al. [13], Cordero et al. [12], and Andreu et al. [14] reported a lower IC_50_ for rapanone. However, the activity against Caco-2 reported by Kuete et al. [13] was comparable to our results. Park et al. [40] demonstrated that the IC_50_ value for embelin was higher than 200 μM after 24 h and then after 48 it was 91.6 μM. This large difference may be the result of different study conditions, as Park’s team used the EZ-CyTox kit to investigate cell viability [40]. Doxorubicin exhibited cytotoxicity toward control cells at the same or much higher level compared to cancer cell lines. Embelin was slightly safer, as it was less or similarly toxic to that of both control and cancer cells. In our previous study, similar relationships were observed with respect to melanoma and keratinocyte cell lines [11].

Data from the current study revealed for the first time the in vitro antiprostate and antithyroid cancer activity of rapanone. Furthermore, rapanone was highly active against an aggressive type of prostate cancer, PC3. Additionally, it seems to be simultaneously selective toward cancerous cells and less toxic against normal cells. Our conclusion on the beneficial selectivity profile is consistent with the conclusions of some other researchers [13,14].

## 4. Materials and Methods

### 4.1. Standards and Reagents

The standards of embelin (98% pure) and rapanone (98% pure) were obtained from Sigma-Aldrich and Sequoia Research Products, respectively. HPLC purity grade acetonitryl, chloroform, orthophosphoric acid, and deionized water were obtained from Chempur Poland. Doxorubicin, cell culture medium (Dulbecco’s Modified Eagle Medium/Nutrient Mixture F-12, DMEM/F12, Dulbecco’s Modified Eagle Medium low glucose, DMEM low glucose, Modified Eagle Medium, MEM with non-essential amino acids NEAA), Triton X-100, penicillin-streptomycin solution, foetal bovine serum FBS, sodium phosphate buffer (pH 7.0) were purchased from Sigma-Aldrich. All reagents used were of analytical grade. The LDH viability test was purchased from Clontech.

### 4.2. Plant Material

Plant specimens of the *Ardisia crenata* Sims white-berried variety from Van den Bos Premium Ardisia’a B.V. (Steenenburchweg 11, s-Gravenzande, The Netherlands) were purchased in a local florist store (Świat Roślin, Cracow, Poland). Botanical identification was performed by Dr. E. Skrzypczak-Pietraszek of the Department of Pharmaceutical Botany, Jagiellonian University Medical College, Cracow, Poland. The voucher sample (ACRE-W-2017) is deposited in the Department of Pharmacognosy, Jagiellonian University Medical College, Cracow, Poland. *Lysimachia punctata* L. was collected from controlled cultivation in The Garden of Medicinal Plants, Jagiellonian University, Cracow, Poland (GPS coordinates: latitude 50.011298, longitude 19.994175). The voucher specimen (KFg/2010031) is deposited in the Department of Pharmacognosy, Pharmaceutical Faculty, Medical College, Jagiellonian University, Cracow, Poland.

### 4.3. Optimization of Extraction Conditions and Preparation of Samples and Study Design

Aliquots of 0.2 g finely ground leaves of *A. crenata* Sims were transferred to round bottom glass flasks. For extraction by each of the three methods, each sample was poured with 20 mL of solvent. To choose the best extractant for the tested plant material, three solvents, differing in polarity, were chosen: chloroform, ethyl acetate, and acetone. Three time levels of the processes were implemented. HRE and SE were conducted for 30, 60, and 120 min, while the UAE was conducted for 10, 20, and 30 min. To study the influence of the number of extraction repetitions on its efficacy, another parameter was introduced, and samples were single- (one portion of the solvent), double- (the sample was twice extracted, every time with a new portion of the solvent), or triple-extracted (the sample was three times extracted, every time with a new portion of the solvent). Heat reflux extraction (HRE) was performed in a water bath (90 °C), shaking (SE), and ultrasonic-assisted extractions (UAE), at room temperature. The ultrasonic bath (a Polsonic Palczyński Sp. J., Sonic—3 type) was used for the UAE. The power of the device was 310 W, while the frequency was 50 Hz. Maceration with stirring (SE) was conducted with a laboratory shaker type 358S at a speed of 150 c.p.m. Each combination of experimental parameters was tested in six replicates.

Combinations of parameters with corresponding codes are presented in Table 1.

### 4.4. Quantitative HPLC Analysis

The procedure was carried out with reference to Podolak and Strzałka [2]. All prepared extracts were transferred to 10 mL volumetric flasks, then the solutions were filtered through 0.45 μm membrane filters into the HPLC 1.5 mL vials and the rapanone content was assessed with an HPLC Dionex apparatus equipped with a Hypersil BDS C-18 column, with mobile phase water with 0.1% *v/v* H_3_PO_4_ (A) and acetonitrile (B), A:B 10:90, a column temperature of 25 °C, a flowrate of 1 mL/min, and a detection wavelength of 286 nm. A rapanone calibration curve was determined with the following concentrations of the substance: 1.0, 0.5, 0.25, 0.125, and 0.0625 mg/mL. Based on the rapanone content in the extracts, its amount in dried plant material was calculated and shown in milligrams of rapanone per 1 g of dried weight of leaves of white-berried *A. crenata*. Representative chromatograms are provided in “Supplementary Materials” (Figure S1).

### 4.5. Extraction, Isolation, and Identification of the Benzoquinones

For cytotoxic testing, rapanone and embelin were isolated from leaves of the white-berried variety of *A. crenata* Sims and the roots of *L. punctata L.*, respectively. The procedure was described by Wróbel-Biedrawa et al. [11] and Podolak et al. [46]. The purity and identity were checked by the LC–MS method compared to authentic standards, as we previously showed [11]. NMR spectra are provided in Supplementary Materials (Figures S2–S5).

### 4.6. Cell Culture and In Vitro Cytotoxic Assay

In the study, three human cancer and normal cell lines were used. They were grouped as follows: prostate panel (prostate carcinoma Du145, derived from the metastatic site: brain, ATCC HTB-81; grade IV prostate carcinoma, PC3, derived from the metastatic site: bone, ATCC CRL-1435; prostate epithelial cells PNT2, ECACC 95012613), gastrointestinal panel (colorectal adenocarcinomas Caco2, ATCC HTB-37, and HT29, ATCC HTB-38; hepatocellular carcinoma HepG2, ATCC HB-8065), thyroid panel (follicular thyroid carcinoma FTC133, ECACC 94060901; undifferentiated thyroid carcinoma 8505C, ECACC 94090184). Cells were grown at 37 °C in a 5% CO_2_ atmosphere, with relative humidity, with culture medium DMEM/F12 (PC3, PNT2, FTC133, 8505C, HT29, and HepG2), DMEM low glucose (Du145), MEM with NEAA (Caco-2), supplemented with 10% fetal bovine serum (FBS), and appropriate antibiotics. Cells were seeded in 96-well plates (1.5 × 10^4^ cells/well) and kept for 24 and 48 h. Subsequently, the culture medium was replaced with a fresh medium containing different concentrations of the tested substances from 4 to 50 µg/mL and the cells were incubated for 24 and 48 h. As a reference drug, doxorubicin was used. Cell viability was examined using the LDH assay, as previously described [46,47]. The absorbance was measured at 490 nm (the reference wavelength 600 nm) with a Biotek Synergy microplate reader. Each experiment was performed in triplicate. Cytotoxicity of the samples was measured using the formula:

$$\%\mathrm{cytotoxicity}=\frac{\mathrm{Asample}-\mathrm{Aspont}}{\mathrm{Amax}-\mathrm{Aspont}}\times 100$$

where:

A_sample_ is the absorbance value for cells treated with the tested substances
A_spont_ is the value for spontaneous LDH release
A_max_ the value in cells lysed in the presence of Triton X100
The half-maximal effective concentration value IC_50_, defined as the substance concentration necessary to obtain 50% of dead cells, was determined.

### 4.7. Statistical Analysis

In the study evaluating optimization extraction conditions, the outlying values of rapanone amount in each set of parameters were removed. Thirty-two outlier values were determined using “Extreme Outlier” software (MP System Co., Kraków, Poland), with an algorithm implemented by Shoemaker and based on a robust technique proposed by Tukey [48]. The ANOVA procedure was used to assess the impact of changing individual parameters on the amount of rapanone obtained in various extraction systems. The same statistical procedure was used to determine the regression coefficients of the primary parameters in the respective multinomial models (describing the relationship between the amount of rapanone and the experimental conditions) and to establish the statistical significance of these coefficients. Then, the results obtained for all combinations of parameters were compared using a Kruskal–Wallis test with a Dunn’s posthoc test (by GraphPad 5 Prism, demo version) to identify the results differing significantly from the rest sets of parameters. The results with the highest number of differences were chosen as representing the combinations of parameters, (i.e., the particular variants of experimental systems), that were used giving the highest efficacy of the extraction process. IC_50_ values in cytotoxicity analysis were estimated using GraphPad 5 Prism. A comparison of the means in a cytotoxicity analysis was performed using one-way ANOVA followed by a posthoc Tukey test.

Differences with *p* < 0.05 were considered statistically significant.

## 5. Conclusions

The most efficient extraction of rapanone from the leaves of white-berried *A. crenata* Sims can be achieved with the UAE method, using ethyl acetate or chloroform. However, the method is time-, solvent-, and energy-sparing, and requires special resources. The SE and HRE methods are more time-consuming and require more solvents, but they can be simpler alternatives. For the first time, a high cytotoxic potential of rapanone with a simultaneous beneficial safety profile against prostate cancer cell lines, Du145 and PC3, and thyroid cancer cell lines, FTC133 and 8505C, was revealed. 

Our study has provided further confirmation of the anti-cancer activity of rapanone and its potential for further development as a lead compound in the search for antitumor agents. Pre-clinical studies require access to substantial amounts of pure compounds. The optimal conditions for the extraction of rapanone from the leaves of white-berried *A. crenata* Sims. that were proposed in this study may be helpful to obtain the best yield of this bioactive compound and aid further exploration of its antitumor potential.

## Figures and Tables

**Table 1:** Experimental design and codes for each set of parameters.

Set Code	HRE			Set Code	SE			Set Code	UAE		
	Solvent	Time of Extraction [min]	Number of Extraction Repetitions		Solvent	Time of Extraction [min]	Number of Extraction Repetitions		Solvent	Time of Extraction [min]	Number of Extraction Repetitions
**1H**	chloroform	30	1	**1S**	chloroform	30	1	**1U**	chloroform	10	1
**2H**	chloroform	30	2	**2S**	chloroform	30	2	**2U**	chloroform	10	2
**3H**	chloroform	30	3	**3S**	chloroform	30	3	**3U**	chloroform	10	3
**4H**	chloroform	60	1	**4S**	chloroform	60	1	**4U**	chloroform	20	1
**5H**	chloroform	60	2	**5S**	chloroform	60	2	**5U**	chloroform	20	2
**6H**	chloroform	60	3	**6S**	chloroform	60	3	**6U**	chloroform	20	3
**7H**	chloroform	120	1	**7S**	chloroform	120	1	**7U**	chloroform	30	1
**8H**	chloroform	120	2	**8S**	chloroform	120	2	**8U**	chloroform	30	2
**9H**	chloroform	120	3	**9S**	chloroform	120	3	**9U**	chloroform	30	3
**10H**	ethyl acetate	30	1	**10S**	ethyl acetate	30	1	**10U**	ethyl acetate	10	1
**11H**	ethyl acetate	30	2	**11S**	ethyl acetate	30	2	**11U**	ethyl acetate	10	2
**12H**	ethyl acetate	30	3	**12S**	ethyl acetate	30	3	**12U**	ethyl acetate	10	3
**13H**	ethyl acetate	60	1	**13S**	ethyl acetate	60	1	**13U**	ethyl acetate	20	1
**14H**	ethyl acetate	60	2	**14S**	ethyl acetate	60	2	**14U**	ethyl acetate	20	2
**15H**	ethyl acetate	60	3	**15S**	ethyl acetate	60	3	**15U**	ethyl acetate	20	3
**16H**	ethyl acetate	120	1	**16S**	ethyl acetate	120	1	**16U**	ethyl acetate	30	1
**17H**	ethyl acetate	120	2	**17S**	ethyl acetate	120	2	**17U**	ethyl acetate	30	2
**18H**	ethyl acetate	120	3	**18S**	ethyl acetate	120	3	**18U**	ethyl acetate	30	3
**19H**	acetone	30	1	**19S**	acetone	30	1	**19U**	acetone	10	1
**20H**	acetone	30	2	**20S**	acetone	30	2	**20U**	acetone	10	2
**21H**	acetone	30	3	**21S**	acetone	30	3	**21U**	acetone	10	3
**22H**	acetone	60	1	**22S**	acetone	60	1	**22U**	acetone	20	1
**23H**	acetone	60	2	**23S**	acetone	60	2	**23U**	acetone	20	2
**24H**	acetone	60	3	**24S**	acetone	60	3	**24U**	acetone	20	3
**25H**	acetone	120	1	**25S**	acetone	120	1	**25U**	acetone	30	1
**26H**	acetone	120	2	**26S**	acetone	120	2	**26U**	acetone	30	2
**27H**	acetone	120	3	**27S**	acetone	120	3	**27U**	acetone	30	3

HRE—heat reflux extraction, SE—shaking extraction, UAE—ultrasound-assisted extraction.

**Table 2:** The means (±standard deviation, SD) and medians of rapanone content in the extracts obtained in various experimental systems, calculated as milligrams of rapanone per 1 g of dried weight of plant material.

Set Code	HRE		Set Code	SE		Set Code	UAE	
	Mean [mg/g]	Median [mg/g]		Mean [mg/g]	Median [mg/g]		Mean [mg/g]	Median [mg/g]
**1H**	1.69 ± 0.17	1.70	**1S**	2.23 ± 0.14	2.24	**1U**	17.42 ± 1.38	17.29
**2H**	1.86 ± 0.20	1.92	**2S**	2.75 ± 0.03	2.76	**2U**	17.93 ± 0.51	18.01
**3H**	1.72 ± 0.15	1.70	**3S**	2.45 ± 0.35	2.54	**3U**	19.77 ± 1.30	20.15
**4H**	1.70 ± 0.32	1.61	**4S**	2.32 ± 0.28	2.22	**4U**	20.54 ± 0.91	20.18
**5H**	1.73 ± 0.30	1.71	**5S**	2.83 ± 0.33	2.75	**5U**	19.48 ± 1.10	19.25
**6H**	1.83 ± 0.33	1.74	**6S**	3.06 ± 0.32	2.93	**6U**	17.54 ± 1.22	17.19
**7H**	2.15 ± 0.21	2.16	**7S**	2.30 ± 0.14	3.06	**7U**	20.09 ± 1.63	21.11
**8H**	2.53 ± 0.71	2.37	**8S**	2.99 ± 0.27	3.03	**8U**	17.16 ± 1.63	16.86
**9H**	2.00 ± 0.20	2.01	**9S**	3.28 ± 0.47	3.20	**9U**	17.86 ± 0.54	17.88
**10H**	11.39 ± 0.90	11.52	**10S**	11.76 ± 1.08	11.82	**10U**	12.21 ± 3.09	12.30
**11H**	11.16 ± 5.08	9.37	**11S**	18.42 ± 3.86	19.27	**11U**	12.99 ± 3.79	11.82
**12H**	10.33 ± 1.86	10.80	**12S**	17.79 ± 0.22	17.85	**12U**	13.06 ± 1.39	13.29
**13H**	13.18 ± 0.45	13.28	**13S**	7.16 ± 0.93	7.05	**13U**	21.39 ± 1.21	21.06
**14H**	14.10 ± 1.74	13.91	**14S**	11.90 ± 1.08	12.24	**14U**	19.40 ± 3.41	19.36
**15H**	12.92 ± 1.97	12.39	**15S**	12.76 ± 1.89	12.75	**15U**	14.74 ± 2.29	14.70
**16H**	14.80 ± 1.65	15.09	**16S**	8.11 ± 1.25	8.68	**16U**	18.28 ± 1.37	18.04
**17H**	14.18 ± 1.27	14.46	**17S**	6.43 ± 1.28	6.86	**17U**	18.71 ± 2.46	17.99
**18H**	17.00 ± 2.67	16.42	**18S**	8.34 ± 2.69	8.41	**18U**	10.71 ± 2.13	10.77
**19H**	2.68 ± 1.05	2.43	**19S**	0.05 ± 0.05	0.06	**19U**	3.34 ± 0.31	3.27
**20H**	2.79 ± 1.56	2.83	**20S**	0.25 ± 0.27	0.15	**20U**	2.92 ± 0.66	3.07
**21H**	2.75 ± 0.88	2.26	**21S**	0.02 ± 0.01	0.02	**21U**	2.18 ± 0.71	2.23
**22H**	2.05 ± 0.80	2.10	**22S**	0.13 ± 0.13	0.07	**22U**	0.04 ± 0.05	0.01
**23H**	4.40 ± 2.46	4.12	**23S**	0.14 ± 0.06	0.117	**23U**	0.10 ± 0.09	0.04
**24H**	2.24 ± 0.85	2.55	**24S**	Nd	Nd	**24U**	Nd	Nd
**25H**	2.50 ± 0.81	2.14	**25S**	0.11 ± 0.06	0.12	**25U**	0.17 ± 0.12	0.11
**26H**	1.97 ± 1.22	1.61	**26S**	0.01 ± 0.02	0.000	**26U**	0.01 ± 0.01	0.00
**27H**	1.35 ± 0.77	1.49	**27S**	Nd	Nd	**27U**	0.10 ± 0.09	0.09

Nd—not determined; plant material—leaves of white-berried *A. crenata* Sims.

**Table 3:** IC_50_ values of rapanone, embelin, and doxorubicin in prostate cancer panel.

	IC_50_ [μg/mL]					
Compound	PC3		Du145		PNT2	
	24 h	48 h	24 h	48 h	24 h	48 h
Rapanone	6.50	4.92	7.68	5.07	16.72	12.65
Embelin	9.27	5.24	8.02	4.18	10.14	4.61
Doxorubicin	>50.00	NE	3.18	NE	1.38	NE

NE—not examined; PC3 grade IV prostate carcinoma, derived from metastatic site: bone, ATCC CRL-1435, Du145 prostate carcinoma, derived from metastatic site: brain, ATCC HTB-81; PNT2 prostate epithelial cells, and ECACC 95012613.

**Table 4:** IC_50_ values of rapanone, embelin, and doxorubicin in colorectal carcinoma panel.

	IC_50_ [μg/mL]					
Compound	Caco-2		HT29		HepG2	
	24 h	48 h	24 h	48 h	24 h	48 h
Rapanone	8.79	5.66	16.91	11.67	36.27	16.70
Embelin	6.12	3.37	24.70	13.72	15.98	6.77
Doxorubicin	3.44	NE	1.53	NE	1.03	NE

NE—not examined; Caco2 colorectal adenocarcinoma, ATCC HTB-37, HT29 colorectal adenocarcinoma, ATCC HTB-38, HepG2 hepatocellular carcinoma, and ATCC HB-8065.

**Table 5:** IC_50_ values of rapanone, embelin, and doxorubicin in thyroid cancer panel.

	IC50 [μg/mL]			
Compound	FTC133		8505C	
	24 h	48 h	24 h	48 h
Rapanone	6.01	4.42	7.84	5.50
Embelin	10.51	6.48	18.86	13.84
Doxorubicin	4.02	NE	>40.00	NE

NE—not examined; follicular thyroid carcinoma FTC133, ECACC 94060901; undifferentiated thyroid carcinoma 8505C and ECACC 94090184.

## Data Availability

Data are available on request from the authors.

## Supplementary Materials

The following supporting information can be downloaded at: https://www.mdpi.com/article/10.3390/molecules27227912/s1, Table S1. Significant differences between pairs of medians of rapanone content in dried plant material. Figure S1. Representative HPLC chromatograms showing content of rapanone in extracts from leaves of white-berried *A. crenata* Sims, obtained by different methods (HRE, SE, UAE). Figure S2. ^1^H NMR data for rapanone. Figure S3. ^13^C NMR data for rapanone. Figure S4. ^1^H NMR data for embelin. Figure S5. ^13^C NMR data for embelin.

## Sample Availability

Samples of the compounds are not available.
