# Supplementary material for: Optimization of Extraction Conditions and Cytotoxic Activity of Rapanone in Comparison to Its Homologue, Embelin
Source: Molecules. 2022 Nov 16;27(22):7912. doi: 10.3390/molecules27227912 (PMC9693145; doi:10.3390/molecules27227912)
Supplement: Supplementary file 1 [file molecules-27-07912-s001.zip › molecules-2000024-supplementary.pdf]

Optimization of Extraction Conditions and Cytotoxic Activity of Rapanone in Comparison to Its Homologue, Embelin

Dagmara Wróbel-Biedrawa [1], Agnieszka Galanty [1], Paweł Zagrodzki [2] and Irma Podolak [1,*]

[1] Department of Pharmacognosy, Pharmaceutical Faculty, Medical College, Jagiellonian University, Medyczna 9, 30-688 Cracow, Poland
[2] Department of Food Chemistry and Nutrition, Pharmaceutical Faculty, Medical College, Jagiellonian University, Medyczna 9, 30-688 Cracow, Poland
* Correspondence: irma.podolak@uj.edu.pl

Contact details:

Dagmara Wróbel-Biedrawa: Orcid ID: 0000-0001-9780-7924

Agnieszka Galanty: Orcid ID: 0000-0001-5636-8646

Paweł Zagrodzki: Orcid ID: 0000-0002-5101-036X

Irma Podolak: irma.podolak@uj.edu.pl¸ Orcid ID: 0000-0002-1251-7192

Content:

Table S1. Significant differences between pairs of medians of rapanone content in dried plant material. The medians are encoded according to the codes of extracts that they refer to (Table 1).

Figure S1. Representative HPLC chromatograms showing content of rapanone in extracts from leaves of white-berried *A. crenata* Sims, obtained by different methods (HRE, SE, UAE). Details are provided in "Materials and methods" section in the main manuscript.

Figures S2-S5. ^{1}H NMR and ^{13}C NMR data for rapanone and embelin. NMR spectra were recorded by means of the JEOL spectrometer at 500 MHz (JNM-ECZR500 RS1, JOEL Ltd., Tokyo, Japan) in $CDCl_3$.

^{1}H NMR ($CDCl_3$, 500.13 MHz) δ ppm: 7.6 (2H, bs, 2xOH), 5.9 (1H, s, H of the ring), 2.45 (2H, t, CH_2), 1.48 (2H, t, CH_2), 1.26 (24H, bs,CH_2 x12), 0.87 (3H, t, CH_3)

^{13}C NMR ($CDCl_3$, 125.77 MHz) δ ppm: 169.0 – not visible, which is characteristic for 1,4-dihydroxybenzoquinones, 117.1 (C-3 of the ring), 102.2 (C-6 of the ring), 31.9 – 14.0 (signals from carbons of the side chain)

Table S1. Significant differences between pairs of medians of rapanone content in dried plant material.

Significantly different pairs of medians	Level of significance
18H *vs* 19S	*
8H *vs* 21S	*
18H *vs* 26S	*
18H *vs* 22U	*
18H *vs* 26U	*
11S *vs* 19S	*
11S *vs* 21S	*
11S *vs* 22S	*
11S *vs* 26S	**
11S *vs* 22U	*
11S *vs* 26U	*
12S *vs* 19S	*
12S *vs* 21S	*
12S *vs* 22S	*
12S *vs* 26S	*
12S *vs* 22U	*
12S *vs* 26U	*
19S *vs* 1U	*
19S *vs* 3U	**
19S *vs* 4U	**
19S *vs* 5U	**
19S *vs* 6U	*
19S *vs* 7U	**
19S *vs* 8U	*
19S *vs* 13U	**
19S *vs* 14U	**
19S *vs* 16U	*
19S *vs* 17U	**
20S *vs* 3U	*
20S *vs* 4U	*
20S *vs* 5U	*
20S *vs* 7U	*
20S *vs* 13U	*
20S *vs* 14U	*

20S *vs* 17U	*
21S *vs* 1U	*
21S *vs* 3U	**
21S *vs* 4U	**
21S *vs* 5U	**
21S *vs* 6U	*
21S *vs* 7U	**
21S *vs* 8U	*
21S *vs* 13U	**
21S *vs* 14U	**
21S *vs* 16U	*
21S *vs* 17U	**
22S *vs* 3U	*
22S *vs* 4U	**
22S *vs* 5U	*
22S *vs* 6U	*
22S *vs* 7U	**
22S *vs* 8U	*
22S *vs* 13U	**
22S *vs* 14U	**
22S *vs* 16U	*
22S *vs* 17U	*
23S *vs* 3U	*
23S *vs* 4U	*
23S *vs* 5U	*
23S *vs* 7U	*
23S *vs* 13U	*
23S *vs* 14U	*
23S *vs* 17U	*
25S *vs* 3U	*
25S *vs* 4U	*
25S *vs* 5U	*
25S *vs* 7U	*
25S *vs* 13U	*
25S *vs* 14U	*
25S *vs* 17U	*
26S *vs* 1U	*
26S *vs* 2U	*
26S *vs* 3U	**
26S *vs* 4U	**
26S *vs* 5U	**
26S *vs* 6U	**
26S *vs* 7U	**
26S *vs* 8U	**
26S *vs* 9U	*

26S *vs* 13U	**
26S *vs* 14U	**
26S *vs* 15U	*
26S *vs* 16U	**
26S *vs* 17U	**
1U *vs* 22U	*
1U *vs* 26U	*
2U *vs* 22U	*
2U *vs* 26U	*
3U *vs* 22U	**
3U *vs* 23U	*
3U *vs* 25U	*
3U *vs* 26U	**
3U *vs* 27U	*
4U *vs* 22U	**
4U *vs* 23U	*
4U *vs* 25U	*
4U *vs* 26U	**
4U *vs* 27U	*
5U *vs* 22U	**
5U *vs* 23U	*
5U *vs* 25U	*
5U *vs* 26U	**
5U *vs* 27U	*
6U *vs* 22U	**
6U *vs* 26U	*
7U *vs* 22U	**
7U *vs* 23U	*
7U *vs* 25U	*
7U *vs* 26U	**
7U *vs* 27U	*
8U *vs* 22U	*
8U *vs* 26U	*
9U *vs* 22U	*
9U *vs* 26U	*
13U *vs* 22U	**
13U *vs* 23U	**
13U *vs* 25U	*
13U *vs* 26U	**
13U *vs* 27U	**
14U *vs* 22U	**
14U *vs* 23U	*
14U *vs* 25U	*
14U *vs* 26U	**
14U *vs* 27U	*

16U *vs* 22U	*
16U *vs* 26U	*
17U *vs* 22U	**
17U *vs* 23U	*
17U *vs* 25U	*
17U *vs* 26U	**
17U *vs* 27U	*

* $p < 0.05$, ** $p < 0.01$

To compare the results obtained for all combination of parameters a Kruskal-Wallis test with a Dunn's post-hoc test were performed. 78 combinations were compared, and the medians varied significantly at level p<0.0001, while Kruskal-Wallis statistic for the whole set of combinations was 377.3.

The medians are encoded according to the codes of extracts that they refer to. Codes are presented in Materials and Methods in the main body of manuscript (Table 5).

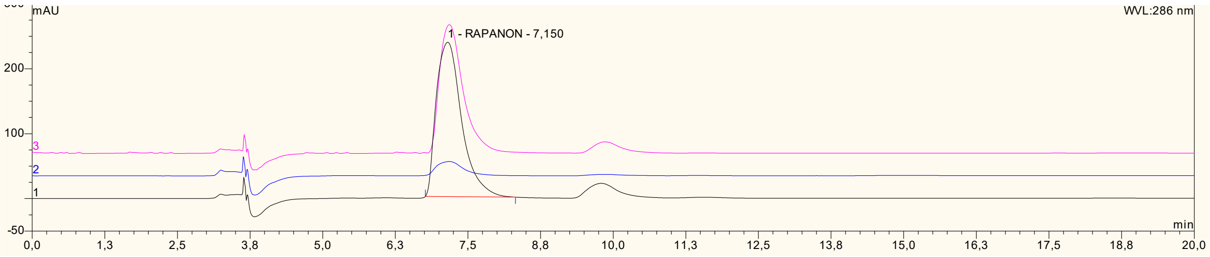

Figure S1. Representative HPLC chromatograms showing content of rapanone in extracts from leaves of white-berried *A. crenata* Sims, obtained by different methods (HRE, SE, UAE).

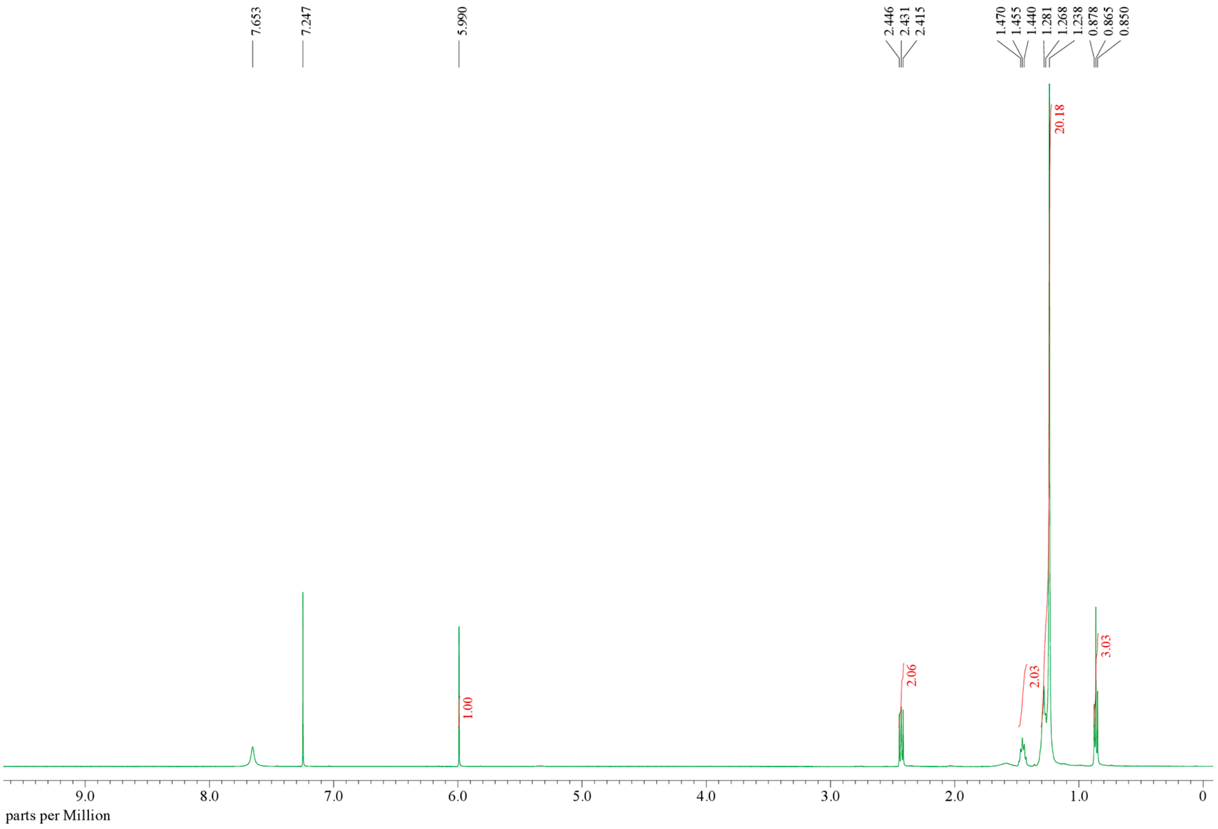

Figure S2. ^{1}H NMR data for rapanone.

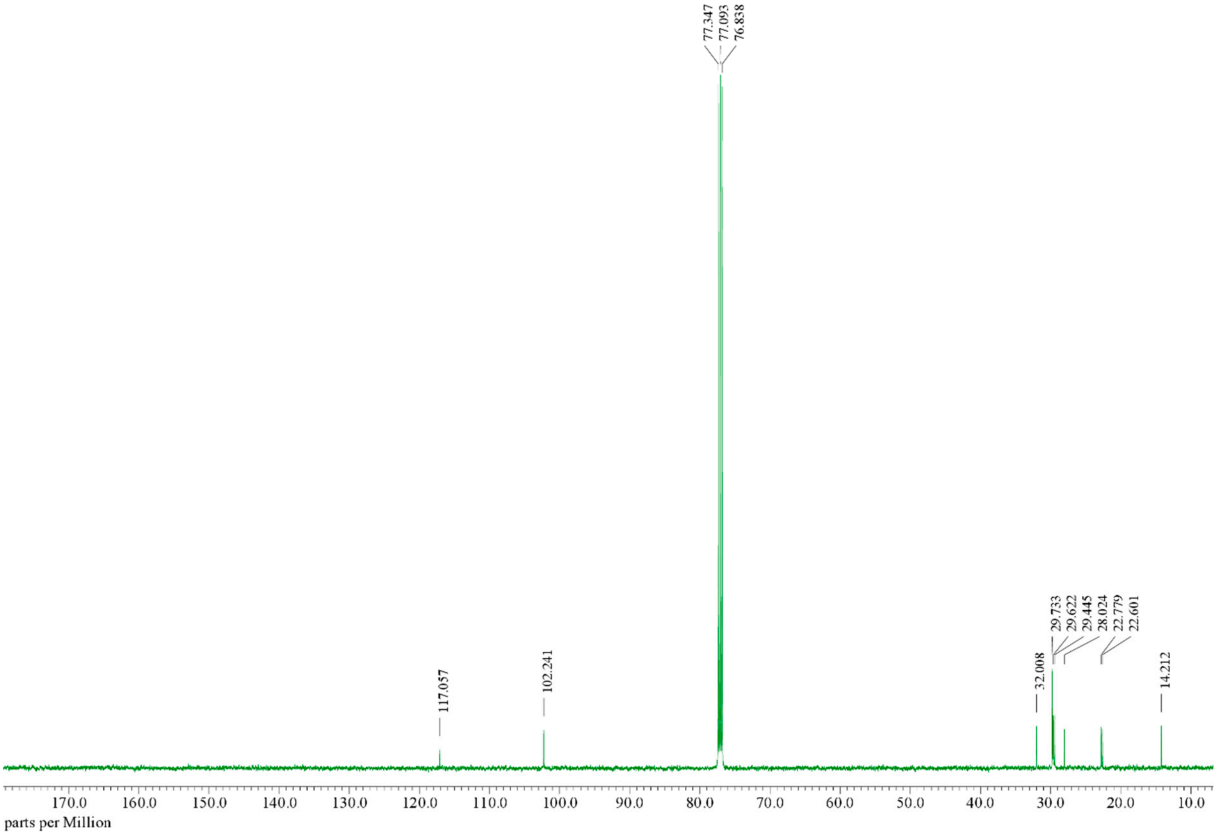

Figure S3. ^{13}C NMR data for rapanone.

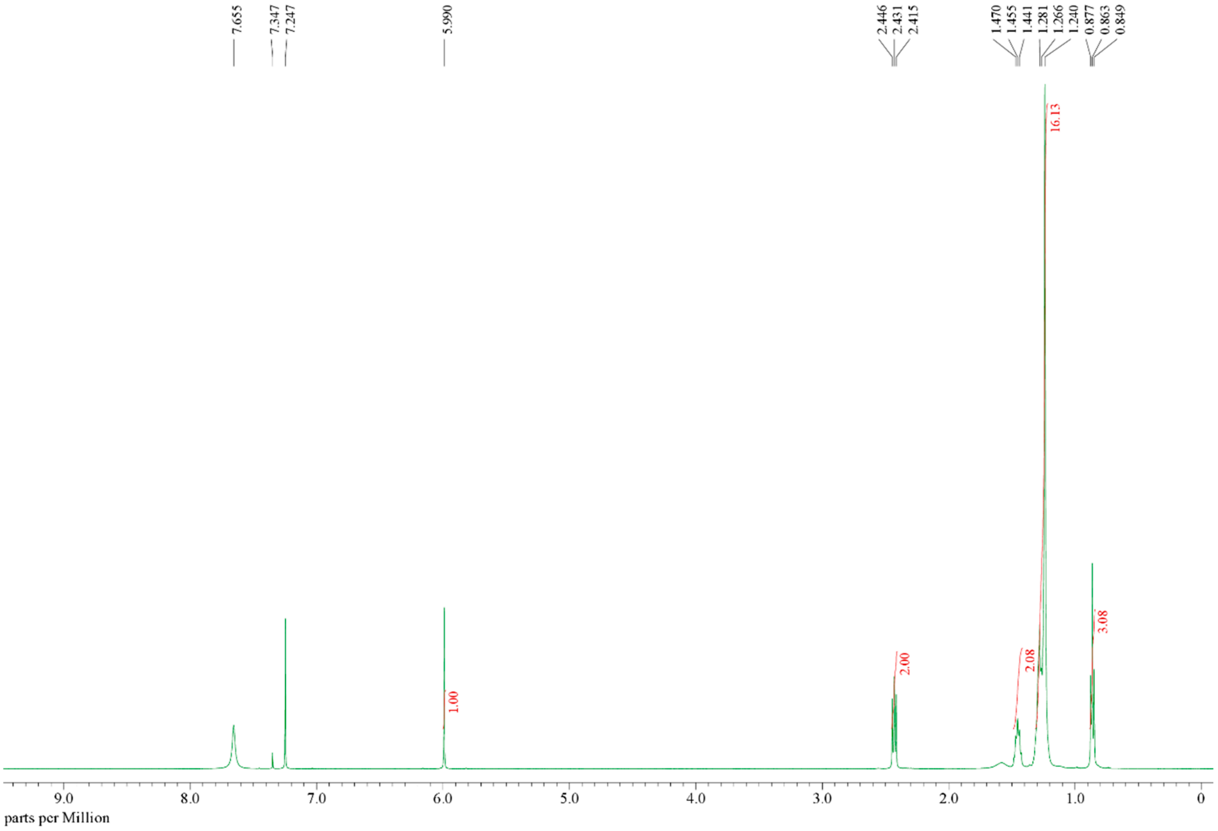

Figure S4. ^{1}H NMR data for embelin.

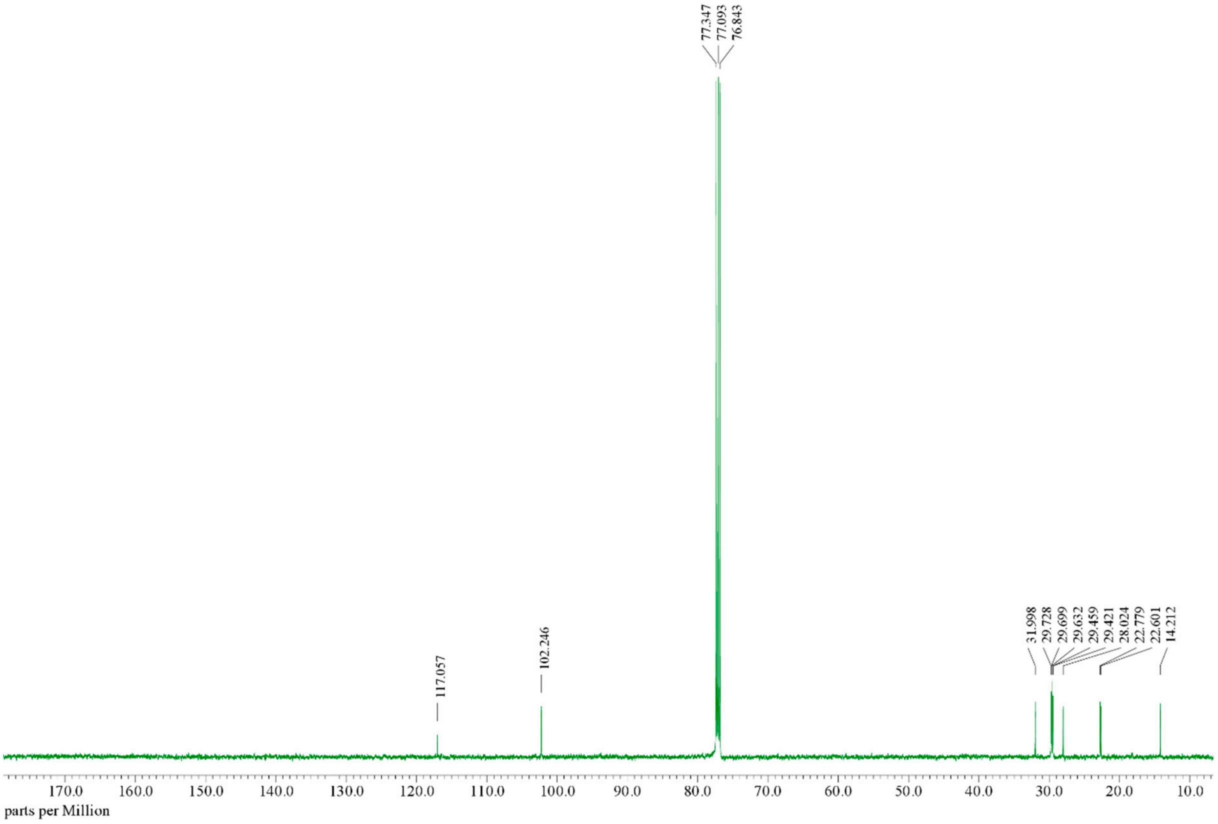

Figure S5. ^{13}C NMR data for embelin.
